# Legal Notes for Institutional Workers

**Published:** 1912-04-13

**Authors:** 


### LEGAL NOTES FOR INSTITUTIONAL WORKERS.

**(The Editor will be pleased to answer Legal queries in this column.)**

### Cremation and Murder.

A gruesome but nevertheless important question was touched upon by Sir Charles Cameron at a recent meeting of the Cremation Society. He drew attention to an argument advanced by counsel for the defence in a recent murder case. "If," said the advocate, "my client had committed this murder, would he not have had the body of his victim cremated?" Like many another argument, this is plausible until the true facts with regard to cremation are revealed. A dead body cannot be thus disposed of unless certain stringent formalities of the law are complied with. He who is minded to cremate some dear relation or friend must needs answer questions on oath, and his application must be accompanied by two medical certificates, in one of which the medical man has to testify not only that he had attended the deceased in the last illness, but that he had seen and identified the body after death. He must also answer searching questions as to the cause of death, and he must certify that there was no circumstance known to him which can give rise to any form of suspicion.

### Necessary Proofs for Compensation.

In the recent appeal case brought by the Manchester Scarlet Fever Hospital from a County Court decision for compensation to be paid to a porter under the Compensation Act, the Master of the Rolls said: "A workman cannot recover compensation unless he can satisfy the Court that there was a particular time, place, and that there were particular circumstances in which the injury by accident happened." He went on to point out that it was not sufficient to say that among many places and times where and when the fever might have been contracted, this occasion of cleaning the mortuary was the most likely. He also pointed out that if County Court judges were to draw inferences upon insufficient evidence, the consequences would be serious, and hospital authorities would be liable for any disease contracted by any of their staff in performance of their duties. In thus deciding this case the Court of Appeal has laid down no new principle; they have merely applied an old principle to a new set of facts, and have applied it in a manner which is eminently satisfactory to those whose servants are *ex necessitati* constantly exposed to infection. One recalls the case of a man who, while engaged in a sewer, inhaled sewer-gas and contracted enteritis, which is not one of the industrial diseases specially mentioned in the Act. The Court held that he could not recover compensation, as it was impossible to say when, where, and how the disease was contracted.

### The Discretion of the Surgeon.

It will be long before the medical profession in this country forgets the lesson of the *cause célèbre* in which a prominent London gynæcologist was sued a few years ago by a patient on whom he had performed oöphorectomy. That lesson has imbued British surgeons with a keen appreciation of the necessity of obtaining consent before witnesses for any contemplated procedure. A not quite parallel, but instructive, case has lately been the basis of a lawsuit in New York. A woman was advised to consult a certain surgeon for appendicitis. The surgeon, after examining her, explained that a definite diagnosis was difficult, and proposed to operate through an incision which would give him an opportunity of inspecting the pelvic organs as well as the appendix. The patient gave the surgeon a free hand to do as he thought best. An inflamed and adherent appendix was found and removed, and the appendages of the right side were freed from adhesions, but were left intact. It was then discovered that the left ovary was the seat of a suppurative process, so it was removed through a separate incision. The patient convalesced speedily and safely, but brought an action for "trespass and assault" on the strength of the separate incision. *Mala praxis* was not alleged. The damages claimed were ten thousand dollars, but fortunately the surgeon was vindicated and the suit failed.

